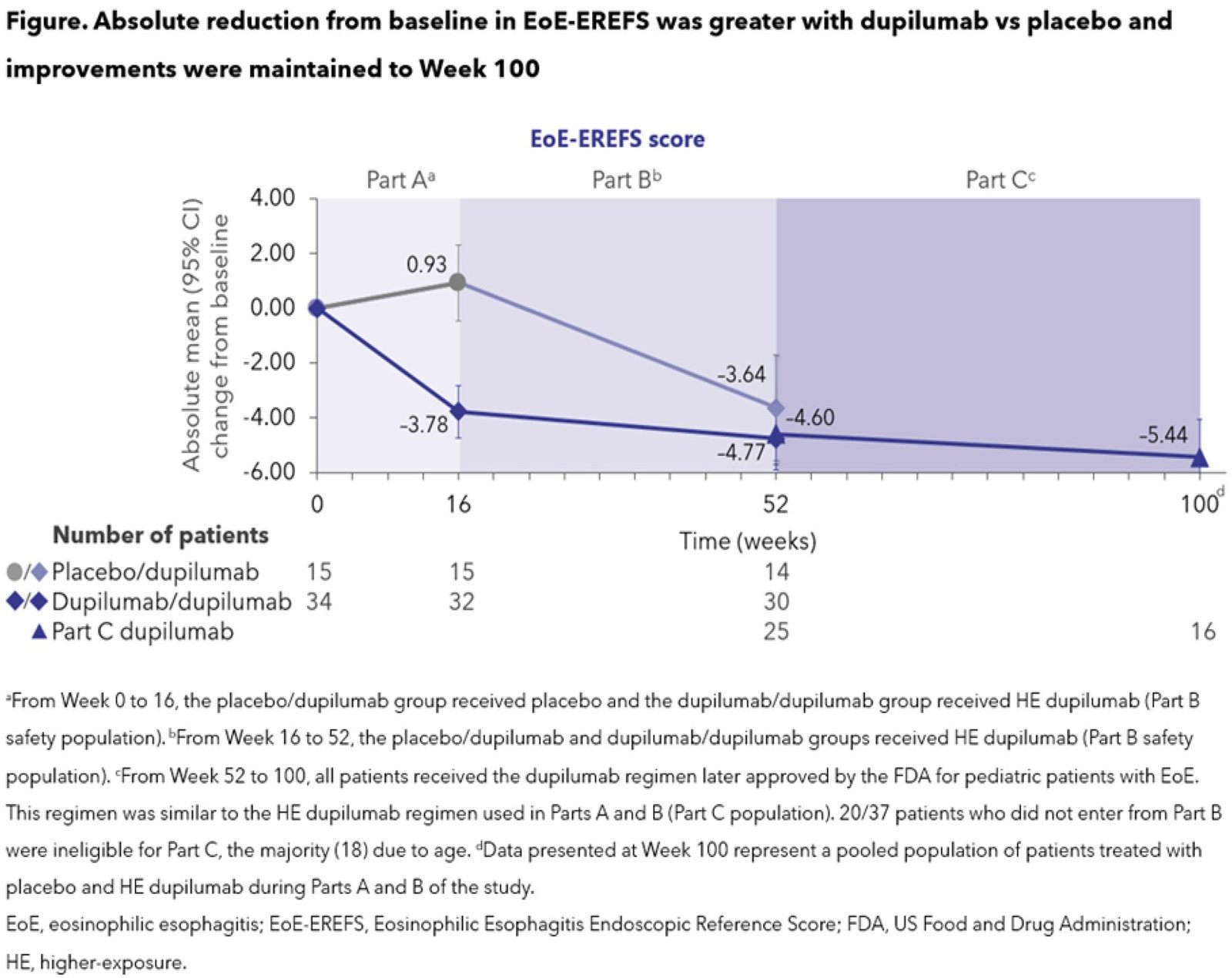# Poster Session II - A196 LONG-TERM DUPILUMAB MAINTAINS HISTOLOGIC AND ENDOSCOPIC IMPROVEMENTS IN CHILDREN WITH EOSINOPHILIC ESOPHAGITIS (EOE): 100-WEEK DATA FROM THE OPEN-LABEL EXTENSION (OLE) OF THE EOE KIDS STUDY

**DOI:** 10.1093/jcag/gwaf042.196

**Published:** 2026-02-13

**Authors:** M Chehade, E Dellon, R D Pesek, M H Collins, D Ashok, R AlKhouri, R Liu, M Louisias, A Radin

**Affiliations:** Mount Sinai Center for Eosinophilic Disorders, Icahn School of Medicine at Mount Sinai, New York, NY; Center for Esophageal Diseases and Swallowing, University of North Carolina School of Medicine, Chapel Hill, NC; Department of Pediatrics, Division of Allergy/Immunology, University of Arkansas for Medical Sciences and Arkansas Children’s Hospital, Little Rock, AR; Division of Pathology and Laboratory Medicine, Department of Pediatrics, Cincinnati Children’s Hospital Medical Center and Department of Pathology & Laboratory Medicine, University of Cincinnati College of Medicine, Cincinnati, OH; Children’s Hospital, London Health Sciences Centre, Western University, London, ON, Canada; Sanofi, Toronto, ON, Canada; Regeneron Pharmaceuticals Inc., Tarrytown, NY; Sanofi, Cambridge, MA; Regeneron Pharmaceuticals Inc., Tarrytown, NY

## Abstract

**Background:**

In EoE KIDS (NCT04394351), dupilumab significantly improved histologic and endoscopic outcomes vs placebo at Week (W)16 (Part A) in patients with EoE aged 1–11 years, with benefits maintained up to W52 (Part B).

**Aims:**

We assessed the efficacy and safety of long-term dupilumab in the EoE KIDS OLE (Part C).

**Methods:**

Patients completing W52 of Part B could enter Part C where they received the weight-tiered, open-label dupilumab regimen subsequently approved by the FDA (similar to the Part A/B higher-exposure regimen). Efficacy data, which included patients who received placebo or dupilumab higher-exposure during Parts A and B, are reported to the last prespecified assessment at W100; safety data included all patients.

**Results:**

102, 98, and 61 patients enrolled in Parts A, B, and C, respectively. At W52, 69.7% (23/33) of patients who received dupilumab achieved ≤6 eosinophils/high-power field (eos/hpf) and this was maintained at W100 (68.2% [15/22]) in Part C. Similar results were reported for <15 eos/hpf (81.8% [27/33] and 90.9% [20/22] at W52 and W100, respectively). Mean changes (95% confidence interval) from Part A baseline at W52 and W100 were similar for EoE-Histologic Scoring System grade/stage scores (–0.87 [–1.01, –0.73] and –0.85 [–1.04, –0.65]/–0.83 [–0.96, –0.71] and –0.87 [–1.04, –0.70]) and EoE-Endoscopic Reference Score (–4.60 [–5.71, –3.49] and –5.44 [–6.83, –4.05]) (Figure). Mild-to-moderate adverse events (AEs) were reported in 86.9% (53/61) of patients during Part C; injection-site reaction was the most common treatment-related AE. Serious AEs were reported in 4.9% (3/61) of patients, with none deemed related to dupilumab.

**Conclusions:**

Dupilumab maintained histologic and endoscopic efficacy in pediatric patients with EoE up to W100 and demonstrated a consistent safety profile.

**Funding Agencies:**

Sanofi and Regeneron Pharmaceuticals Inc.